# An Improved Genetically Encoded Red Fluorescent Ca^2+^ Indicator for Detecting Optically Evoked Action Potentials

**DOI:** 10.1371/journal.pone.0039933

**Published:** 2012-07-10

**Authors:** Masamichi Ohkura, Takuya Sasaki, Chiaki Kobayashi, Yuji Ikegaya, Junichi Nakai

**Affiliations:** 1 Brain Science Institute, Saitama University, Saitama, Japan; 2 Laboratory of Chemical Pharmacology, Graduate School of Pharmaceutical Sciences, University of Tokyo, Tokyo, Japan; University G. D’Annunzio, Italy

## Abstract

Genetically encoded Ca^2+^ indicators (GECIs) are powerful tools to image activities of defined cell populations. Here, we developed an improved red fluorescent GECI, termed R-CaMP1.07, by mutagenizing R-GECO1. In HeLa cell assays, R-CaMP1.07 exhibited a 1.5–2-fold greater fluorescence response compared to R-GECO1. In hippocampal pyramidal neurons, R-CaMP1.07 detected Ca^2+^ transients triggered by single action potentials (APs) with a probability of 95% and a signal-to-noise ratio >7 at a frame rate of 50 Hz. The amplitudes of Ca^2+^ transients linearly correlated with the number of APs. The expression of R-CaMP1.07 did not significantly alter the electrophysiological properties or synaptic activity patterns. The co-expression of R-CaMP1.07 and channelrhodpsin-2 (ChR2), a photosensitive cation channel, in pyramidal neurons demonstrated that R-CaMP1.07 was applicable for the monitoring of Ca^2+^ transients in response to optically evoked APs, because the excitation light for R-CaMP1.07 hardly activated ChR2. These technical advancements provide a novel strategy for monitoring and manipulating neuronal activity with single cell resolution.

## Introduction

Monitoring activities of individual neurons is crucial for the understanding of neuronal circuit dynamics. For this purpose, Ca^2+^ imaging of neurons with fluorescent Ca^2+^ indicators is a promising technique, because neuronal action potentials (APs) evoke Ca^2+^ transients that are relatively large and easy to detect. Genetically encoded Ca^2+^ indicators (GECIs) (or fluorescent Ca^2+^ indicator proteins, FCIPs) are a recent alternative to chemically synthesized fluorescent Ca^2+^ indicators, such as Fura-2 and Oregon Green BAPTA-1 [1], [2]. GECIs provide several remarkable advantages over synthetic indicators. First, GECIs are applicable to mature neurons; in general, the loading efficiency of synthetic indicators in neurons both *in vitro* and *in vivo* decreases as the age of preparation increases [3]. Second, GECIs can target specific cell types and subcellular compartments [4]–[7]. Third, GECIs are stably expressed once the genes are introduced into cells, which allows for the long-term recording of neuronal activity from the same cells over weeks and months [8]. These advantages of GECIs have been widely applied to Ca^2+^ imaging in viable cells in various model animals, such as mouse, nematode, fly and zebrafish [8]–[13].

However, popularly used green fluorescent protein (GFP)-based GECIs, such as G-CaMPs [14] and Cameleons [15], are not simply applied to cells that express light-gated non-selective cation channels for photostimulation of neurons, such as channelrhodopsin-2 (ChR2) [16], because the excitation light for the indicators also activates the light-gated channels. Thus, in order to independently control the light-gated channels during the excitation of Ca^2+^ indicators, it is necessary to devise means such as to use different laser powers for Ca^2+^ imaging and photostimulation [17].

**Figure 1:**
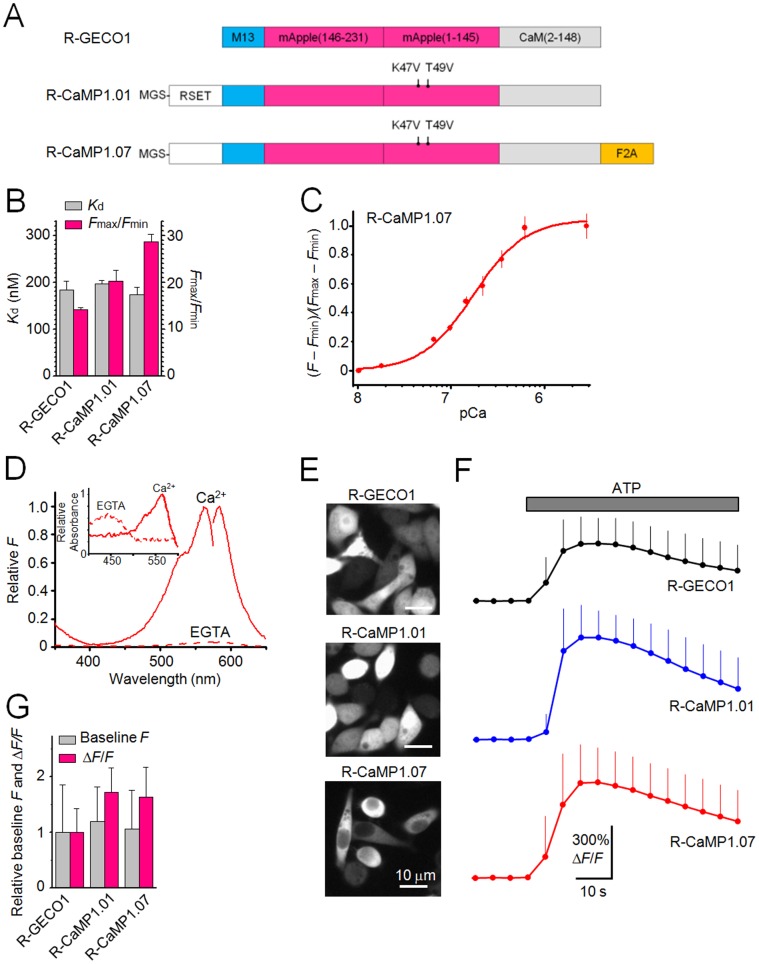
Characterization of R-CaMPs *in vitro* and in HeLa cells. **A**, Schematic structures of R-CaMPs. Mutations are indicated with respect to R-GECO1. RSET and M13 are a tag that encodes hexahistidine and a target peptide for a Ca^2+^-bound CaM derived from MLCK, respectively. The amino-acid numbers of mApple and CaM are indicated in parentheses. **B**, Ca^2+^ affinity (*K*_d_) and dynamic range (*F*_max_/*F*_min_). Error bars, s.d. (*n* = 3 each). **C**, Ca^2+^ titration curve. Curves were fit according to the Hill equation. The *K*_d_ is shown in **B**. Error bars, s.d. (*n* = 3 each). **D**, Normalized fluorescence and absorbance (inset) spectra of R-CaMP1.07 in 1 µM Ca^2+^ or 1 mM EGTA. **E**, Fluorescence images of HeLa cells expressing red fluorescent GECIs. **F**, Mean Δ*F*/*F* responses to the application of 100 µM ATP in HeLa cells. Error bars, s.d. (*n* = 163 cells for R-GECO1, 182 cells for R-CaMP1.01 and 166 cells for R-CaMP1.07). **G**, Baseline fluorescence and peak responses (Δ*F*/*F*) to the application of 100 µM ATP in HeLa cells. Error bars, s.d.

Zhao et al [18] recently developed a red fluorescent Ca^2+^ indicator, R-GECO1, in which a circularly permuted red fluorescent protein (mApple) is linked to calmodulin (CaM) and its target peptide M13 from myosin light chain kinase at its C- and N-termini, respectively (Fig. 1A). However, the usefulness of R-GECO1 as a novel tool for detection of Ca^2+^ signals in response to neuronal APs has not been demonstrated.

We report a novel red fluorescent GECI, R-CaMP1.07, which improved R-GECO1. R-CaMP1.07 exhibited superior performance to R-GECO1 in HeLa cells and hippocampal pyramidal neurons. R-CaMP1.07 detected Ca^2+^ signals associated with single APs with 95% probability. We also demonstrated that R-CaMP1.07 is the first GECI applicable for combinatorial use with ChR2; Ca^2+^ signals in response to APs evoked by the photostimulation of ChR2 were detected by R-CaMP1.07.

## Results

### Development of an Improved Red Fluorescent GECI, R-CaMP1.07

To create superior red fluorescent GECIs, we introduced random mutations into a prototype molecule, R-GECO1 [18] with the RSET tag at the N-terminus. The variants were screened *in vitro*, and the best variant R-CaMP1.01, with K47V and T49V mutations in the circularly permuted mApple domain, was identified (Fig. 1A). R-CaMP1.01 exhibited a ∼1.4-fold greater increase in fluorescence following exposure to saturating Ca^2+^ (*F*_max_/*F*_min_ = 20.3±2.28, *n* = 3) compared to R-GECO1 (*F*_max_/*F*_min_ = 14.2±0.44, *n* = 3) (Figs. 1B, C). Since it was reported that the GECI functionality of G-CaMP2, our previous green fluorescent GECI, was modified by peptide fusion [5], we further tested if such fusion of several peptides with R-CaMP1.01 at the N- or C-terminus would modify the functionality of R-CaMP1.01 and anticipated that some variants would be superior to R-CaMP1.01. As a result, we found a variant of R-CaMP1.01 termed R-CaMP1.07 (Fig. 1A), which had a self-cleaving peptide, F2A [19] at the C-terminus of R-CaMP1.01, exhibited the largest Ca^2+^-dependent fluorescence change (*F*_max_/*F*_min_ = 28.7±1.59, *n* = 3), which was ∼2-fold greater than R-GECO1 (Fig. 1B). The affinity of R-CaMP1.01 and R-CaMP1.07 for Ca^2+^ did not differ significantly from R-GECO1 (Fig. 1B). The Ca^2+^ titration curve for R-CaMP1.07 is shown in Figure 1C. The spectra of R-CaMP1.07 were similar to those of R-GECO1; the absorbance and emission peaks were 562 nm and 584 nm, respectively, in the presence of Ca^2+^ (Fig. 1D).

To assess the functionality of GECIs in a cellular environment, R-CaMPs and R-GECO1 were expressed in HeLa cells. R-GECO1 and R-CaMP1.01 localized not only to the cytoplasm but also to nucleus, whereas R-CaMP1.07 localized only to the cytoplasm (Fig. 1E). The baseline fluorescence of R-CaMP1.07 was similar to that of R-GECO1, but the fluorescence response of R-CaMP1.07 to ATP stimulation (Δ*F*/*F*) was 1.5–2-fold greater than that of R-GECO1 (Figs. 1F and G).

### Comparison of R-CaMP1.07 and R-GECO1 in Brain Slices

**Figure 2:**
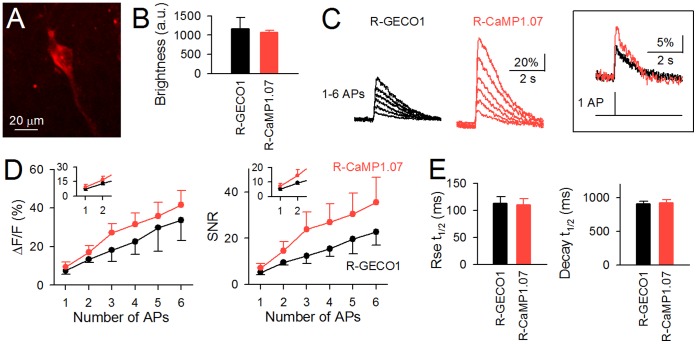
Comparison of the performance of R-GECO1 and R-CaMP1.07 in hippocampal pyramidal cells. **A**, A confocal image showing a CA3 pyramidal cell expressing R-CaMP1.07 in a cultured slice. **B**, Mean fluorescent intensities of neurons expressing R-GECO1 and R-CaMP1.07. Error bars, s.e.m. (*n* = 6 cells each). *P*>0.05, Student’s *t*-test. **C**, Representative Δ*F*/*F* traces in response to trains of 1–6 APs delivered at 50 Hz for a pyramidal cell expressing R-GECO1 (left) and R-CaMP1.07 (right). The same traces for 1 AP are magnified in the right panel. **D**, Mean Δ*F*/*F* response and SNR plotted against the number of APs in R-GECO1 (black) and R-CaMP1.07 (red). Insets are magnified views of 1–2 APs. Error bars, s.e.m. (*n* = 5 cells each). **E**, The mean rise and decay time constant calculated from Ca^2+^ transients evoked by single APs. Error bars, s.e.m. (*n* = 5 cells each). *P*>0.05, Student’s *t*-test.

We next characterized the performance of R-CaMP1.07 and R-GECO1 expressed in rat hippocampal CA3 pyramidal neurons in cultured slices. The red fluorescent GECIs were expressed in the neurons via targeted single-cell electroporation of their expression plasmids [20]. Robust expression of the GECIs was observed 24–48 h after electroporation (Fig. 2A). The baseline fluorescence of R-CaMP1.07 did not significantly differ from that of R-GECO1 (Fig. 2B; *P*>0.05, Student’s *t*-test). Trains of APs at a frequency of 50 Hz were induced by current injection into the neurons, and the AP-induced Ca^2+^ transients were imaged from the soma and proximal dendrites at 50 frames per second (fps) using a Nipkow-disk confocal microscope. All experiments were performed under identical experimental conditions at room temperature (25–28°C). R-CaMP1.07 detected the Ca^2+^ transients associated with single APs with 100% probability in 7 out of 8 tested cells. In total, R-CaMP1.07 and R-GECO1 detected Ca^2+^ transients in response to single APs with 95% and 70% probability, respectively. The detection probability of Ca^2+^ transients evoked by >2 APs was 100% for both R-CaMP1.07 and R-GECO1. The Δ*F*/*F* of Ca^2+^ transients evoked by single APs were 9.4±2.6% and 7.4±1.7% for R-CaMP1.07 and R-GECO1, respectively, and the signal-to-noise ratios (SNRs) were 7.2±1.9 and 5.2±1.1 at 50 fps, respectively (Figs. 2C and D; *n* = 5 each). Both indicators exhibited an almost linear increase in Δ*F*/*F* and SNRs up to 6 APs. Over the entire stimulus range, Δ*F*/*F* and SNRs of R-CaMP1.07 were consistently 1.5–2.0-fold higher than those of R-GECO1. The rise and decay time constants of the AP-induced Ca^2+^ transients were nearly identical between R-CaMP1.07 and R-GECO1 (*P*>0.05, Student’s *t*-test) (Figs. 2D and E).

**Figure 3:**
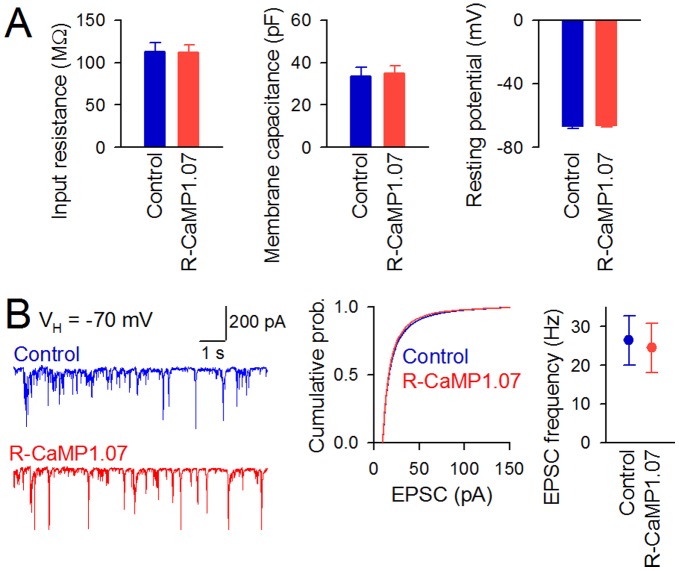
Electrophysiological properties of hippocampal neurons expressing R-CaMP1.07. **A**, Average input resistance (left), membrane capacitance (middle), or resting potential (right) did not significantly differ between control and R-CaMP1.07 groups. Error bars, s.e.m. (*n* = 7 cells each). *P*>0.05, Student’s *t*-test. **B**, Representative spontaneous EPSCs recorded from a control and R-CaMP1.07-expressing cell at a holding potential of −70 mV (left). Cumulative probability of amplitudes of EPSCs is shown in the middle panel (Control, *n* = 11062 events; R-CaMP1.07, *n* = 10265 events). *P*>0.05, Kolmogorov-Smirnov test. Frequency of EPSCs is shown in the right panel. Error bars, s.e.m. (*n* = 7 cells each). *P*>0.05, Student’s *t*-test.

We next tested electrophysiological properties of neurons expressing R-CaMP1.07. No significant differences in input resistance, membrane capacitance, or resting potential were observed between control (R-CaMP1.07-negative) and R-CaMP1.07-positive cell groups (Fig. 3A). The amplitudes and frequency of spontaneous excitatory postsynaptic currents (EPSCs) in R-CaMP1.07-positive cells were not significantly different from those of control cells (Fig. 3B). These results suggest that R-CaMP1.07 expression *per se* does not cause abnormal changes in cellular electrophysiological properties or synaptic activity.

### Monitoring of Optically Evoked Neuronal Activity with R-CaMP1.07

**Figure 4:**
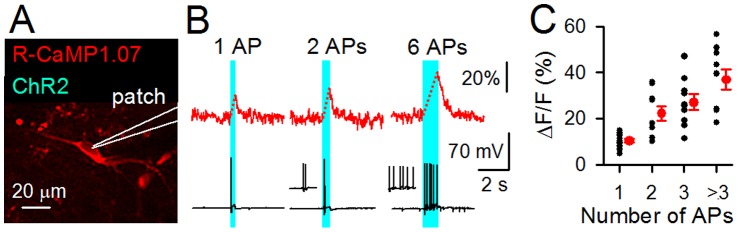
Simultaneous monitoring and manipulation of neuronal activity by co-expression of R-CaMP1.07 and ChR2. **A**, A projection image of a CA3 pyramidal cell expressing R-CaMP1.07 and ChR2. The cell was whole-cell recorded to measure the number of APs. **B**, Imaging of ChR2-triggered-APs by changes in the R-CaMP1.07 fluorescence (top). APs were evoked by a pulse of 470-nm light with a duration of 300 to 3000 ms (blue region indicates time of photostimulation). Putative Ca^2+^ increases during the photostimulation are represented by broken lines (Bottom). The number of APs was recorded using the current-clamp mode. **C**, Δ*F*/*F* amplitude of the R-CaMP1.07 responses as a function of the number of APs induced by photostimulation. The peak Δ*F*/*F* amplitudes were calculated from the first frames after termination of the photostimulation. Individual data are plotted as black dots and their averages are shown in red. Error bars, s.e.m. (*n* = 4 cells).

The excitation wavelength of R-CaMP1.07 ranged from 500 to 580 nm (Fig. 1D), which rarely overlaps with the photostimulation wavelength range of ChR2 [16]. Therefore, it should be possible to use R-CaMP1.07 for monitoring the neuronal activity evoked by photostimulation of ChR2 in an identical cell. We co-expressed R-CaMP1.07 and ChR2 in identical cells to explore this hypothesis (Fig. 4A). Excitation and photostimulation wavelengths of 568 nm and 488 nm were used to image and manipulate neuronal APs, respectively, and the number of APs evoked by photostimulation was confirmed using whole-cell patch clamp recording. Photostimulation with 488 nm light triggered APs and transient increases in R-CaMP1.07 fluorescence (Fig. 4B). However, no changes in membrane potential were induced during the imaging period of R-CaMP1.07 fluorescence with 568 nm light at least up to 10 mW. Therefore, in our imaging conditions, ChR2 was not activated by the 568 nm light. These observations verified that it is possible to independently image and manipulate the neural activity in an identical cell with R-CaMP1.07 and ChR2. Increases in the duration of photostimulation increased the number of APs. The amplitudes of AP-induced Ca^2+^ transients linearly correlated with the number of APs (Fig. 4C), which is similar to the observation in Figure 2D.

## Discussion

In this study, we developed an improved red fluorescent Ca^2+^ indicator, R-CaMP1.07 by introducing mutations to the prototype indicator R-GECO1. In hippocampal neurons, R-CaMP1.07 exhibited 1.5–2-fold greater fluorescence signals than R-GECO1. We also demonstrated that R-CaMP1.07 serves as the first GECI for combinatorial use with ChR2, which is a popular optogenetical tool for neuronal stimulation.

The mutated residues Lys-47 and Thr-49 in R-CaMP1.01 (Fig. 1A) are located in the third β-strand of mApple [21]. Both residues are assumed to be facing outside of the chromophore [22], [23]. Since the substitution of Val-48, which is located between the residues 47 and 49, with Alanine in mKate2 [24], a red fluorescent protein homologous to mApple, has been reported to enhance brightness by improving the protein maturation, the K47V and T49V mutations might have similar steric effects on R-CaMP1.01.

R-GECO1 or R-CaMP1.01 was unexpectedly localized not only in the cytoplasm but also in the nucleus in HeLa cells (Fig. 1E); whereas R-CaMP1.07 localized only in the cytoplams. Interestingly, nuclear entry was hardly observed with any variant of G-CaMPs in our HeLa cell-based assay (data not shown), although it was reported that a small portion (∼8%) of mouse cortical neurons expressing G-CaMP3, which was introduced via *in utero* electroporation, showed the labeled nucleus after a relatively long-term expression of postnatal day 28 [25]. The partial entry of G-CaMP3 into the nucleus is attributed to proteolysis, because it was shown that the labeled nucleus contained the indicator without having the N-terminal hexahistidine tag [25]. Partial proteolysis is a possible mechanism for the entry of R-GECO1 or R-CaMP1.01 into the nucleus. On the other hand, G-CaMPs, unlike R-GECO1, R-CaMP1.01 and R-CaMP1.07, possess nuclear export signal sequence(s) and lack nuclear localization signal sequence(s). Indeed, a leucine-rich nuclear export signal sequence, φxx(x)φxx(x)φxφ (whereφ is either leucine, isoleucine, valine, phenylalanine or methionine and x is any amino-acid residue) [26], was found in G-CaMPs [8], [14], [22], [26]–[27], but this signal sequence was absent in R-GECO1, R-CaMP1.01 and R-CaMP1.07. Thus, in the case of R-GECO1 or R-CaMP1.01, the lack of this signal sequence may permit the indicators to enter into the nucleus. In the present study, we successfully exported R-CaMP1.07 out of the nucleus with the addition of a C-terminal F2A peptide, although the mechanism of this effect is unknown.

GECIs may inherently buffer Ca^2+^ dynamics and interfere with cellular processes, but R-CaMP1.07 expression did not significantly alter cellular electrophysiological properties or synaptic activity patterns under our experimental conditions. The detection of isolated neuronal APs during a single trial is an imperative goal for GECIs in neurophysiology. We demonstrated that R-CaMP1.07 detected the Ca^2+^ transients in response to single APs with a 95% probability in cultured hippocampal neurons. However, one point to note is that we obtained the data at 25–28°C. Since the dynamics of intracellular Ca^2+^ and the sensitivity of Ca^2+^ indicators are affected by temperature, we cannot exclude the possibility that the detectability of R-CaMP1.07 might be changed depending on temperature and preparations. In the future study, R-CaMP1.07 should be evaluated in more physiological experimental systems, such as acute slices at physiological temperature (34–37°C) or *in vivo*, because the fluorescence response of GECIs are apt to become smaller *in vivo* conditions compared to *in vitro* conditions [8], [13].

The rise and decay time constants in R-CaMP1.07-expressing cells were 110 and 920 ms, respectively. These values were still larger than G-CaMPs [8], which exhibit rise time constants of 80–100 ms and decay time constants of 600–700 ms. The rapid kinetics of Ca^2+^ indicators allows imaging with increased temporal resolution, which can separate fast individual APs in burst-spike trains. Further improvement in the kinetics of R-CaMPs will facilitate a more faithful detection of APs.

ChR2 is widely used as a tool to control neuronal excitability in various cell types and tissues [28], [29]. However, ChR2 is not preferably applied for combinatorial use with green fluorescent GECIs (e.g., G-CaMPs [8], [14], [22], [26]–[27]) or chemically synthesized green fluorescent Ca^2+^ indicators (e.g., OGB-1 and fluo-4), because the excitation light for these indicators also activates ChR2 and this makes it difficult to independently manipulate ChR2 during the excitation of indicators. In this study, we demonstrated that our improved red fluorescent GECI, that was excited at a wavelength substantially distinct from the action spectra of ChR2, enabled the imaging of neuronal activity triggered by ChR2. We expect that our understanding of the roles for specific cells in the neuronal network will be promisingly facilitated by the advanced technique for simultaneous visualization and manipulation of neuronal activity with our R-CaMP1.07 and ChR2 in combination.

## Materials and Methods

### Plasmid Construction

Complementary DNA (cDNA) encoding R-CaMP1.01 was synthesized by randomly mutagenizing the cDNA encoding the prototype GECI, R-GECO1 [18] with the RSET tag at the N-terminus, as reported previously [30]. R-CaMP1.07 was generated by fusing a cDNA encoding the F2A peptide [19] to the 3′ end of a cDNA encoding R-CaMP1.01 via a linker encoding the amino-acid sequence GGGTGGSGGGGGGEF (in one-letter code). The cDNAs encoding R-GECO1 and ChR2 were chemically synthesized (GenScript). The cDNAs encoding red fluorescent GECIs were subcloned into a pRSET_B_ vector (Invitrogen) containing a T7 promoter as described previously [30] for bacterial expression or a pEGFP-N1 vector (Clontech) with a CMV promoter, as described previously [14] for expression in HeLa cells and cultured rat hippocampal neurons. The cDNA encoding ChR2 was subcloned into a pEGFP-C1 vector (Clontech). All of the constructs were verified by sequencing.

### Bacterial Protein Expression and *in vitro* Characterization

*E. coli* KRX (Promega) transformed with bacterial expression plasmids for red fluorescent GECIs were grown at 37°C. Protein expression was induced by the addition of 0.1% rhamnose and incubating for an additional 5 h at 20°C. The indicator proteins with N-terminal histidine tags were purified [30], dialyzed against a KM buffer containing (in mM) 100 KCl and 20 MOPS (pH 7.2) and were used for *in vitro* characterization. R-GECO1, which lacks the N-terminal histidine tag, was used without purification. Spectral analyses were performed as described previously [31]. The term “dynamic range” was defined as *F*_max_/*F*_min_, where *F*_max_ is the fluorescence intensity at saturating [Ca^2+^], and *F*_min_ is the fluorescence intensity at nominally zero [Ca^2+^] with 1 mM EGTA. The Ca^2+^ titration experiments were performed at pH 7.2 with 10 mM solutions of K_2_H_2_EGTA and Ca_2_EGTA from Ca^2+^ Calibration Kit #1 (Invitrogen), as reported previously [8].

### Ca^2+^ Imaging in HeLa Cells

HeLa cells were cultured in Dulbecco’s modified Eagle’s medium containing 10% fetal bovine serum and transfected with plasmids using Lipofectamine 2000 (Invitrogen) according to the manufacturer’s manual. The fluorescence images of cells expressing red fluorescent GECIs were acquired using a fluorescence microscope (IX71, Olympus) equipped with a CCD camera (ORCA-ER, Hamamatsu), as described previously [31] except for the use of the excitation filter (BP545–580), the dichroic mirror (DM600) and the emission filter (BA610IF). The cells were perfused with HEPES-buffered saline (HBS) containing (in mM) 135 NaCl, 5.4 KCl, 2 CaCl_2_, 1 MgCl_2_, 10 glucose and 5 HEPES (pH 7.4), and 100 µM ATP was bath-applied for 1 min after the baseline fluorescence was obtained. The images were analyzed using the AquaCosmos version 2.0 software (Hamamatsu). The transient increase in fluorescence (Δ*F*/*F*) was calculated after subtracting the background fluorescence.

### Cultured Slice Preparation and Single-cell Electroporation

All experiments were performed with the approval of the animal experiment ethics committee at the University of Tokyo (approval number: 19–43) and according to the University of Tokyo guidelines for the care and use of laboratory animals. Hippocampal slices were prepared from postnatal day 7 Wistar/ST rats (SLC), as described previously [32]. Briefly, rat pups were chilled with ice and decapitated. The brains were removed and cut horizontally into 300-µm slices using a DTK-1500 vibratome (Dosaka) in aerated, ice-cold Gey’s balanced salt solution supplemented with 25 mM glucose. The entorhino-hippocampal stumps were excised and cultivated on Omnipore membrane filters (JHWP02500, Millipore), which were laid on plastic O-ring disks [33]. The cultures were incubated in a humidified incubator at 37°C in 5% CO_2_ with 1 ml of 50% minimal essential medium, 25% Hanks’ balanced salt solution (HBSS), 25% horse serum (Cell Culture Laboratory) and antibiotics. The medium was changed every 3.5 days. On days 3–5 *in vitro*, R-CaMP1.07 or R-GECO1, under the control of the CMV promoter, were introduced into neurons via targeted single-cell electroporation. Briefly, borosilicate glass pipettes (tip resistance 5–7 MΩ) were filled with HBSS containing 1–2 µg/µl of plasmid DNA and 200 µM Alexa Fluor 488 hydrazide (Invitrogen). The tip of the pipette was placed in close proximity to the soma, and electroporation was performed with 50 rectangular pulses (–5 V, 0.5 ms duration) at a frequency of 50 Hz [20]. The single-cell electroporation was applied sequentially to a maximum of 20 cells using the same pipette within 10 min. Imaging was performed 24–48 h after electroporation.

### Electrophysiology and Ca^2+^ Imaging in Cultured Hippocampal Slices

Hippocampal slices were mounted in a recording chamber and perfused at a rate of 1.5–3 ml/min with aCSF containing (in mM) 127 NaCl, 26 NaHCO_3_, 3.3 KCl, 1.24 KH_2_PO_4_, 1.0 MgSO_4_, 1.0 CaCl_2_ and 10 glucose, bubbled with 95% O_2_ and 5% CO_2_. All recordings were performed at room temperature (25–28°C), unless otherwise specified. Patch-clamp recordings were collected from hippocampal CA3 pyramidal neurons using a MultiClamp 700 B amplifier and a Digidata 1440A digitizer controlled by pCLAMP10 software (Molecular Devices). Epifluorescence microscopy was used to select cells containing Alexa Fluor 488 signals with the fluorescence intensity ranging from 50–85 (arbitrary units). Borosilicate glass pipettes (5–7 MΩ) were filled with a solution containing (in mM) 135 K-gluconate, 4 KCl, 10 HEPES, 10 phosphocreatine-Na_2_, 0.3 Na_2_-GTP and 4 Mg-ATP (pH 7.2). The signals were low-pass filtered at 1–2 kHz and digitized at 20–100 kHz. Data were discarded if the access resistance changed by more than 20% during the experiments. Spikes were evoked by current injections (2–3 ms, 1–2 nA). For Ca^2+^ imaging, red fluorescent GECIs were excited at 568 nm (power below objective lens 8.82 µW) with a laser diode (641-YB-A01, Melles Griot) and visualized using a 617-nm (width 73 nm) band-pass emission filter. Images were captured at 50 fps using a Nipkow-disk confocal scanner unit (CSU-X1, Yokogawa Electric), a cooled CCD camera (iXON DV897, Andor), an upright microscope (Eclipse FN1, Nikon) and a water-immersion objective (40 ×, 0.9 numerical aperture, Nikon). For the photostimulation of ChR2-expressing-neurons, a blue light pulse (wavelength 465–495 nm, power below objective lens 5–720 µW) was delivered through the same confocal unit for 300–3000 ms. For data analysis, the cell bodies and proximal dendrites of the neurons were identified visually to locate regions of interest (ROIs). The fluorescence intensity in each ROI was spatially averaged. The fluorescence change was defined as Δ*F*/*F* = (*F_t_* – *F*_0_)/*F*_0_, where *F_t_* is the fluorescence intensity at time *t*, and *F*_0_ is the baseline averaged for 2 s before time *t*. The maximum Δ*F*/*F* within 1 s after the action potential initiation was used as the peak amplitude of the Ca^2+^ transient. The signal-to-noise (S/N) ratio was defined as the average spike signal amplitude divided by the standard deviation of the baseline fluorescence intensity in the trace. Data were collected from more than 3 consecutive trials. The rise time *t*_1/2_ was measured as the time between the onset of the spike initiation and the half-peak response. The decay time *t*_1/2_ was measured as the time of half decay of a single exponential fit of the recovery from the peak response to the baseline.
